# The Anti-Histamine Azelastine, Identified by Computational Drug Repurposing, Inhibits Infection by Major Variants of SARS-CoV-2 in Cell Cultures and Reconstituted Human Nasal Tissue

**DOI:** 10.3389/fphar.2022.861295

**Published:** 2022-06-30

**Authors:** Robert Konrat, Henrietta Papp, Janine Kimpel, Annika Rössler, Valéria Szijártó, Gábor Nagy, Mónika Madai, Safia Zeghbib, Anett Kuczmog, Zsófia Lanszki, Tanja Gesell, Zsuzsanna Helyes, Gábor Kemenesi, Ferenc Jakab, Eszter Nagy

**Affiliations:** ^1^ Department of Structural and Computational Biology, Max Perutz Labs, University of Vienna, Vienna, Austria; ^2^ Calyxha Biotechnologies GmbH, Vienna, Austria; ^3^ National Laboratory of Virology, Szentágothai Research Centre, University of Pécs, Pécs, Hungary; ^4^ Institue of Biology, Faculty of Sciences, University of Pécs, Pécs, Hungary; ^5^ Department of Hygiene, Microbiology and Public Health, Institute of Virology, Medical University of Innsbruck, Innsbruck, Austria; ^6^ CEBINA (Central European Biotech Incubator and Accelerator) GmbH, Vienna, Austria; ^7^ Department of Pharmacology and Pharmacotherapy, Medical School and Szentágothai Research Centre, University of Pécs, Pécs, Hungary

**Keywords:** COVID-19, computational drug repurposing, SARS-CoV-2, azelastine, anti-viral activity, variants of concern, nasal colonization

## Abstract

**Background and purpose:** The COVID-19 pandemic continues to pose challenges, especially with the emergence of new SARS-CoV-2 variants that are associated with higher infectivity and/or compromised protection afforded by the current vaccines. There is a high demand for additional preventive and therapeutic strategies effective against this changing virus. Repurposing of approved or clinically tested drugs can provide an immediate solution.

**Experimental Approach:** We applied a novel computational approach to search among approved and commercially available drugs. Antiviral activity of a predicted drug, azelastine, was tested *in vitro* in SARS-CoV-2 infection assays with Vero E6 cells, Vero cells stably overexpressing the human TMPRSS2 and ACE2 proteins as well as on reconstituted human nasal tissue using the predominant variant circulating in Europe in summer 2020, B.1.177 (D614G variant), and its emerging variants of concern; B.1.1.7 (alpha), B.1.351 (beta) and B.1.617.2 (delta) variants. The effect of azelastine on viral replication was assessed by quantification of viral genomes by droplet digital PCR or qPCR.

**Key results:** The computational approach identified major drug families, such as anti-infective, anti-inflammatory, anti-hypertensive, antihistamine, and neuroactive drugs. Based on its attractive safety profile and availability in nasal formulation, azelastine, a histamine 1 receptor-blocker was selected for experimental testing. Azelastine reduced the virus-induced cytopathic effect and SARS-CoV-2 copy numbers both in preventive and treatment settings upon infection of Vero cells with an EC_50_ of 2.2–6.5 µM. Comparable potency was observed with the alpha, beta and delta variants. Furthermore, five-fold dilution (containing 0.02% azelastine) of the commercially available nasal spray formulation was highly potent in inhibiting viral propagation in reconstituted human nasal tissue.

**Conclusion and Implications:** Azelastine, an antihistamine available as nasal sprays developed against allergic rhinitis may be considered as a topical prevention or treatment of nasal colonization by SARS-CoV-2. A Phase 2 efficacy indicator study with azelastine-containing nasal spray that was designed based on the findings reported here has been concluded recently, confirming accelerated viral clearance in SARS-CoV-2 positive subjects.

## Introduction

The COVID-19 (Coronavirus Disease 2019) pandemic represents a worldwide threat to public health since its emergence, evoking unprecedented global efforts to control it. By the end of 2021, approximately 275 million people have been infected and approximately 5.3 million have died according to official records (Coronavirus Pandemic (COVID-19)—the data - Statistics and Research - Our World in Data, 2021). The major focus has been on prophylaxis through the introduction of mass vaccination, with ∼58% of the world’s population being fully vaccinated. However, higher vaccination coverage is needed to control the pandemic and prevent the overload of health systems, as a consequence of the emergence of new virus variants. The original SARS-CoV-2 virus (designated as Wuhan) has been replaced by variants that are more adapted to the human host, mostly in binding to ACE2, the cellular receptor of the virus, often with higher affinity (Barton et al., 2021; Zhang et al., 2021). The Spike protein (S-protein), the viral protein responsible for this interaction, is the target of current vaccination strategies that in addition to changes that increase the receptor binding affinity, also mutate to avoid natural and vaccine-induced or immune therapy-provided immunity. Therefore, there is still a high medical need for effective treatment options for the disease.

Given the time pressure to develop new anti-COVID approaches, the established model and timelines of drug discovery and development are not appealing and repurposing of approved drugs is considered as an attractive alternative to identify drugs suitable for prevention and/or therapy of COVID-19 (Pushpakom et al., 2018).

Since SARS-CoV-2 initially infects epithelial cells of the nasopharynx, an early anti-viral intervention could be achieved with topical administration of antiviral compounds. This may show benefit both for the individual receiving such treatment by inhibiting progression of disease to the lower airways, and the community by lowering transmission rate and viral spread. Therefore, special interest is focused on the repurposing of nasal spray products, which act either non-specifically by forming a physiochemical barrier for the attachment of the viruses to its host cells or by true antiviral effect. Some of these repurposed nasal drugs have reached the clinical phase of testing against COVID-19 (Figueroa et al., 2021; Winchester et al., 2021).

Here we have applied a novel computational prediction approach using signature mapping with biochemical pathways affected by proven or putative SARS-CoV-2 drugs. Key to our strategy is the poly-pharmacological hypothesis, i.e. that drugs simultaneously interact and interfere with numerous targets and thereby rewire biochemical pathway networks. Drug identification is therefore defined by identifying compounds that match a pre-defined pathway modulation profile.

This prediction approach has identified drug families and approved drugs, some with proven anti-SARS-CoV-2 activity or clinical efficacy. Most importantly, we provide evidence that the histamine 1 (H1) receptor blocker azelastine, widely used in allergic rhinitis therapy in a nasal spray formulation, is effective against SARS-CoV-2 infection in several *in vitro* assays.

## Materials and Methods

### Computational Method to Identify Putative COVID-19 Drugs

The identification strategy described here is based on the hypothesis that drugs with similar biochemical pathway (activity) profiles will address similar disease areas. Our computational approach was based on the Shannon-Entropy Descriptor (SHED) concept in which the chemical structure of the drug is converted into a 2D topological graph where nodes correspond to the atoms in the drug and edges connecting two nodes indicate the existence of a chemical bond. The most important feature of the SHED approach is that seemingly different molecules with significantly different 2D molecular structures can nevertheless have similar Shannon entropy vectors resulting in similar biochemical (pharmacological) activity patterns. Details of the computational approach are given in the Supplementary Material 1.

**FIGURE 1:**
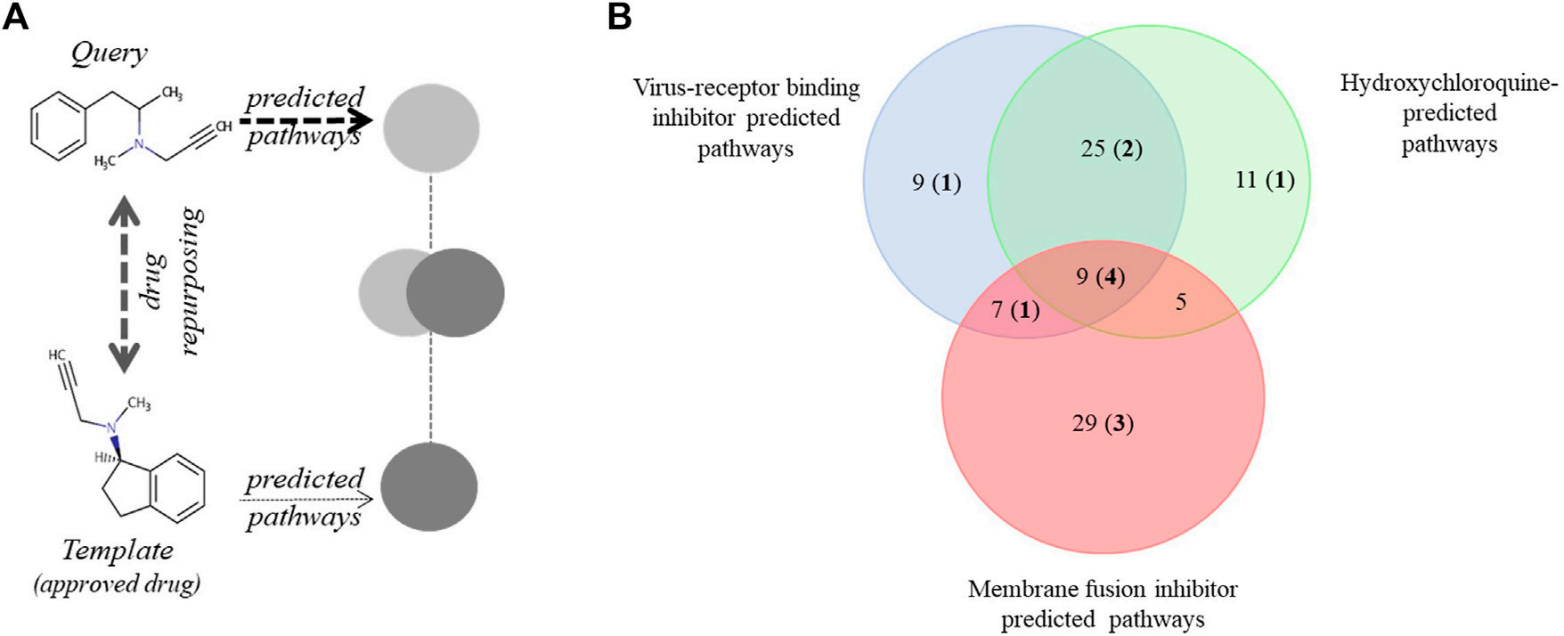
Pathway-based drug repurposing and overlap among three predicted and an experimentally verified pathway data set. **(A)** Illustration of pathway-based drug repurposing. A predefined pathway profile obtained for a query drug (outlined in Supplementary Figure S1) is screened against a data set of template drugs with pre-calculated pathway profiles (for example, clinically approved drugs). Interesting drugs relevant for repurposing applications are obtained *via* maximizing pathway overlap. **(B)** Pathways predicted to be involved in SARS-CoV-2 infection were identified using three drugs shown to be active against SARS-CoV: a virus-receptor binding inhibitor: SSAA09E2, viral and cellular membrane fusion inhibitor: SSAA09E3 and hydroxychloroquine. Numbers in bold and parenthesis indicate the number of pathways also detected in an experimental data set by Zhou et al. (2020). KEGG pathways involved in this analysis are shown in Supplementary Material 2.

The starting point in our search strategy was the selection of a desirable pathway profile and the subsequent search for clinically approved drugs that match this predefined activity pattern (Figure 1A, Supplementary Figure S1). Relevant experimental information was available from a recent analysis of the KEGG pathways involved in SARS-CoV-2 infection (Zhou et al., 2020). Secondly, we employed pathway information we predicted for drugs shown to be active against SARS-CoV and/or SARS-CoV-2. Three experimentally verified and characterized compounds, hydroxychloroquine (Yao et al., 2020), and the SARS-CoV targeting experimental drugs SSAA09E2 {N-[[4-(4-methylpiperazin-1-yl)phenyl]methyl]-1,2-oxazole-5-carboxamide} and SSAA09E3 {N-(9,10-dioxo-9,10-dihydroanthracen-2-yl)benzamide} (Adedeji et al., 2013) were employed. Hydroxychloroquine reduces endosomal acidification, SSAA09E2 acts by blocking early interactions of SARS-CoV with its receptor, the angiotensin converting enzyme 2 (ACE2), shared by SARS-CoV-2 and SSAA09E3 prevents fusion of the SARS-CoV viral membrane with the host cellular membrane. For all three selected ligands, the pathway profiles were calculated and the 50 highest scoring pathways were considered for the analysis.

### Viruses, Cells Culture, Tissue Culture and Compounds

SARS-CoV-2 hCoV-19/Hungary/SRC_isolate_2/2020, Accession ID: EPI_ISL_483637 is representative of the wide-spread European lineage carrying spike protein substitution D614G and was isolated in Hungary. SARS-CoV-2 variants were isolated at the Medical University of Innsbruck. The isolates belong to the B.1.1.7 (alpha, isolate C63.1, EPI_ISL_3277382), B.1.351 (beta, isolate C24.1, EPI_ISL_1123262), B.1.617.2 (delta, isolate D25.1, EPI_ISL_3760186) and B.1.177 (D614G, isolate B86.2, EPI_ISL_3305837) GISAID Clade.

Vero E6 (African green monkey kidney epithelial) cells (ECACC Cat. No. 85020205) were cultured in Dulbeccos`s Modified Eagle Medium (DMEM; Lonza) supplemented with 10% heat inactivated (HI) fetal bovine serum (FBS) (Gibco) and 1% penicillin-streptomycine (Lonza). Vero cells stably overexpressing human serine protease TMPRSS2 and ACE2 receptor were generated as described elsewhere (Riepler et al., 2021) and cultured in DMEM (Merck, Darmstadt, Germany) containing 2% FBS (PAN-Biotech).

Effect of azelastine-HCl on cell viability was assessed with standard viability assays (CellTiter-Glo^®^ Luminescent Cell Viability Assay, Promega, cat#: G7572 or MTT assay).

MucilAir™ human nasal tissue generated from healthy donors was purchased from Epithelix Sarl (Cat#: EP02MP) and maintained according to the producer’s instructions in MucilAir™ culture medium.

Azelastine hydrochloride (azelastine-HCl) was purchased from SelleckChem (S2552) or from Sigma-Aldrich (PHR1636-1G, Lot#LRAC4832) and dissolved in DMSO. Allergodil, the commercially available azelastine-HCl nasal spray was used in tissue culture experiments (0.1% azelastine, Mylan).

### SARS-CoV-2 Infection Assay in Vero E6 Cell Line

Vero E6 cells were seeded on 96-well plates at 4.5×10^4^ cells/well and used at approximately 90% confluence. Azelastine-HCl (SelleckChem) was used at concentrations ranging from 0.4 to 12.5 µM. In a preventive setting, immediately after adding azelastine containing cell culture medium, the cells were infected with SARS-CoV-2 hCoV-19/Hungary/SRC_isolate_2/2020 at an MOI of 0.01. After 30 min incubation in a humidified atmosphere of 5% CO_2_ at 37°C, the culture medium was removed and replaced with fresh culture medium containing azelastine at given concentrations. In the post-infection treatment setting, cells were infected for 30 min without azelastine, then the culture medium containing the virus was removed, and replaced with fresh medium with azelastine. 48 h post infection the cytopathogenic effect was evaluated by microscopic observation semi-quantitatively based on Cytopathic Scores (CPS) ranging from 0 to 4; 0: no cytopathic effect, comparable to uninfected control, 4: CPE is as strong as in the infected control. Cytopathic effect was based on the appearance of “holes” in the otherwise confluent, homogenous layer of cells indicating cell death. Cell supernatant was collected for virus quantification with droplet digital PCR analysis. Experiments with the infective SARS-CoV-2 were performed in the BSL4 facility of the Szentágothai Research Centre, University of Pécs, Hungary, according to institutional regulations.

### Infection of the Vero-TMPRSS2/ACE2 Cell Line by SARS-CoV-2 Variants of Concern.

Vero cells stably overexpressing human serine protease TMPRSS2 and ACE2 receptor (Riepler et al., 2021) were seeded on 96-well plates at 10^4^ cells/well the day before infection. Azelastine hydrochloride, (Sigma-Aldrich) was diluted with Dulbeccos`s Modified Eagle Medium (Merck, Darmstadt, Germany) containing 2% FBS to final concentrations ranging from 25 µM to 0.4 µM. Prior to infection of the cells, cell culture supernatant was aspirated and replaced with 50 µl of the Azelastine-HCl dilutions in the preventive (co-administration) setting and 50 µl of medium in the post-infection (therapeutic) setting. Subsequently, cells were infected with 50 µl of SARS-CoV-2 isolates carrying either the spike protein substitution D614G or belonging to the B.1.1.7 (alpha), B.1.351 (beta) or B.1.617.2 (delta) variants at an MOI of 0.01 for 30 min at 37°C. For both experimental settings, the supernatant then was aspirated and replaced by 50 µl fresh medium and 50 µl of the same azelastine concentrations used before, resulting in Azelastine concentrations ranging between 12.5 and 0.2 µM. 48 h post infection, the cytopathic effect was evaluated and supernatant was used to determine RNA copy number by quantitative real-time PCR.

Experiments with SARS-CoV-2 variants were performed at the Institute of Virology at the Medical University of Innsbruck according to institutional regulations.

### Testing the Anti-SARS-CoV-2 Activity of Azelastine Containing Nasal Spray With Reconstituted Human Nasal Tissue.

MucilAir™ human nasal tissue (Epithelix) was infected with SARS-CoV-2 hCoV-19/Hungary/SRC_isolate_2/2020 at MOI of 0.01 on the apical side. After 20 min incubation at 37 °C in 5% CO_2_, the virus containing medium was removed completely. A five-times diluted (in MucilAir™ culture medium) solution of the Allergodil nasal spray (containing a final concentration of 0.02% azelastine, which corresponds to 523.5 µM) was added onto the apical side (200 µl) for 20 min. Following the treatment, the diluted nasal spray was fully removed from the surface of the cells to provide a liquid-air interface and incubated for 24 h. The 20-min treatment with the diluted Allergodil was repeated at 24 and 48 h post infection (hpi). After 24, 48 and 72 hpi, the apical sides of the cells were washed for 15 min with MucilAir™ Culture medium, and the solution was collected to quantify the viral RNA copy number by Droplet digital PCR as described below. The cells were also inspected under an inverted microscope at 48 and 72 hpi.

### Virus Quantification

Total RNA was extracted from the supernatant or from the apical washes of infected Vero E6 cells using Monarch^®^ Total RNA Miniprep Kit (Promega, Cat#: T2010S). For viral copy number quantification droplet digital PCR technology was applied (Bio-Rad Laboratories Inc. QX200 Droplet Digital PCR System). The primers and probes used were specific for the SARS-CoV-2 RdRp gene (Reverse primer: CAR ATG TTA AAS ACA CTA TTA GCA TA, Forward primer: GTG ARA TGG TCA TGT GTG GCG G, Probe: FAM-CAG GTG GAA CCT CAT CAG GAG ATG C-BBQ). For all concentrations at least three replicates were prepared.

From the supernatant of transgenic Vero cells viral RNA copy was determined with quantitative PCR. Briefly, cell culture supernatant was mixed in a 1:1 ratio with DLR buffer (0.5% IGEPAL, 25 mM NaCl in 10 mM Tris-HCl buffer, 15 µl RiboLock RNase Inhibitor (ThermoScientific, 40 U/µl, EO0381) per ml DLR buffer) to isolate the viral RNA. SARS-CoV-2 genome copies were quantified via qPCR using E gene specific primers (ACA GGT ACG TTA ATA GTT AAT AGC GT and ATA TTG CAG CAG TAC GCA CAC A), FAM-labelled probe (FAM-ACA CTA GCC ATC CTT ACT GCG CTT CG-BHQ1) and iTaq Universal Probes One-Step Kit (BioRad, Cat.# 1725141). An in-house produced, *in vitro* transcribed RNA standard (E gene of SARS-CoV-2) was used to quantify qPCR results. Virus-only wells without azelastine were set to 100% and percent inhibition was calculated for each sample. For each azelastine concentrations triplicate measurements were performed, and three independent experiments were done.

### Data Analysis and Statistics

EC_50_ value of azelastine-HCl against each virus was determined with nonlinear regression (log (inhibitor) vs response—variable slope (four parameter)) using GraphPad Prism 8.4.3 (GraphPad Software, San Diego, California United States).

## Results

### Definition of Query Pathway Profiles for Drug Repurposing

We found significant overlap among the three predicted and the experimental SARS-CoV-2 KEGG pathways (Supplementary Material 2 and Figure 1B). The most prominent was the overlap between the SSAA09E2 and Hydroxychloroquine pathways (34 of the 50 best hits). Nine out of 50 pathways were depicted in all three prediction sets, and four of these were also described in the experimental data set published by Zhou et al. (2020). Altogether 12 of the 95 unique pathways in the three predicted sets were described experimentally as well (Supplementary Material 2). The most commonly identified disease areas associated with the predicted pathways were infectious diseases: viral infections (e.g. measles, hepatitis C, EBV, influenza, HSV), parasitic infections (e.g. Chagas, leishmaniasis, trypanosomiasis, malaria), bacterial infections (e.g. tuberculosis, salmonellosis, shigellosis, pertussis, ETEC) or cellular processes involved during infection (e.g. endocytosis, immune cell signaling) (Supplementary Material 2).

### Screening Drugs for Matching Pathways

**FIGURE 2:**
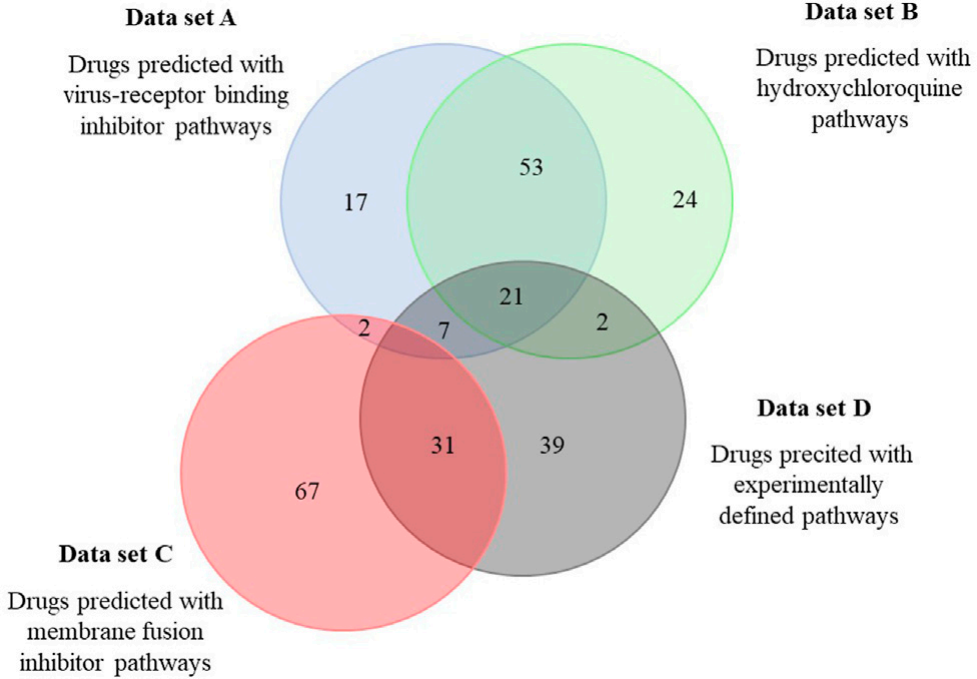
Overlap among drugs identified by the different pathway sets predicted to be involved in SARS-CoV-2 infection. Pathways relevant for SARS-CoV-2 infection predicted to be affected by three selected drugs were used to identify drugs from the DrugBank. Predicted drugs involved in this analysis are shown in Supplementary Material 3.

Next, a set of 2,700 drugs - clinically tested, mostly approved and commercially available via Selleckchem - was screened for matching pathways. Identified hits were stored as an ordered list and ranked based on the number of shared pathways (screened with the 50 best scoring pathways). The 100 top scoring drugs, predicted in four independent screens using the virus-receptor binding inhibitor SSAA09E2 (A), hydoxychloroquine (B), the membrane fusion inhibitor SSAA09E3 (C) and the experimental defined pathways for SARS-CoV-2 (Zhou et al., 2020) (D) were considered for further analysis and are given in Supplementary Material 3 (listing the drugs identified in at least 2 screens and in major drug categories). The overlap between the predicted sets of drugs is in alignment with the corresponding predicted pathways. Approximately half of the drugs predicted based on the pathways of SSAA09E2 or hydoxychloroquine (that show approximately 2/3 overlap) were identical (Figure 2). The main drug classes were anti-infectives (antivirals, antibiotics, antifungals), anti-histamines, anti-inflammatory drugs (non-steroids), anti-hypertensive drugs, diuretics (potassium channel blockers), analgesics and neuroactive drugs (mainly anti-psychotics) (Supplementary Material 3). Approximately 30% of the drugs identified both in data sets A and B were shared with those derived from the pathways from the experimental SARS-CoV-2 data set (D) (21 of 74, Figure 2). We also found a significant drug overlap (approximately 30%) between data sets obtained for the viral fusion inhibitor SSAA09E3 and the experimental SARS-CoV-2 pathways. Here, the overlapping drugs were dominated by steroids of all kinds (anti-inflammatory glucocorticoids, progesterone-analogues).

Anti-histamines were among the most prevalent drugs identified, both H1 and H2 receptor blockers. The H1-blocker anti-allergy medicine, azelastine, was predicted in three independent screens (Supplementary Material 3). Azelastine has no major effect on normal physiology or concerning side-effects, and additionally is available in a nasal spray formulation for topical application and was therefore the focus of further studies.

We predicted genes to be involved in the action of azelastine and hydroxychloroquine and found significant differences in the two gene sets that is indicative of distinct cellular mechanisms that may lead to antiviral effects (Supplementary Material 4). The overlapping genes obtained with azelastine and hydroxychloroquine were mainly related to immune response, which is in line with the anti-inflammatory effects of these two compounds, reported in the literature (Supplementary Material 4) (Ben-Zvi et al., 2012; Watts et al., 2019).

### Anti-SARS-CoV-2 Activity of Azelastine in Vero E6 and Vero-TMPRSS2/ACE2 Cell Lines

**FIGURE 3:**
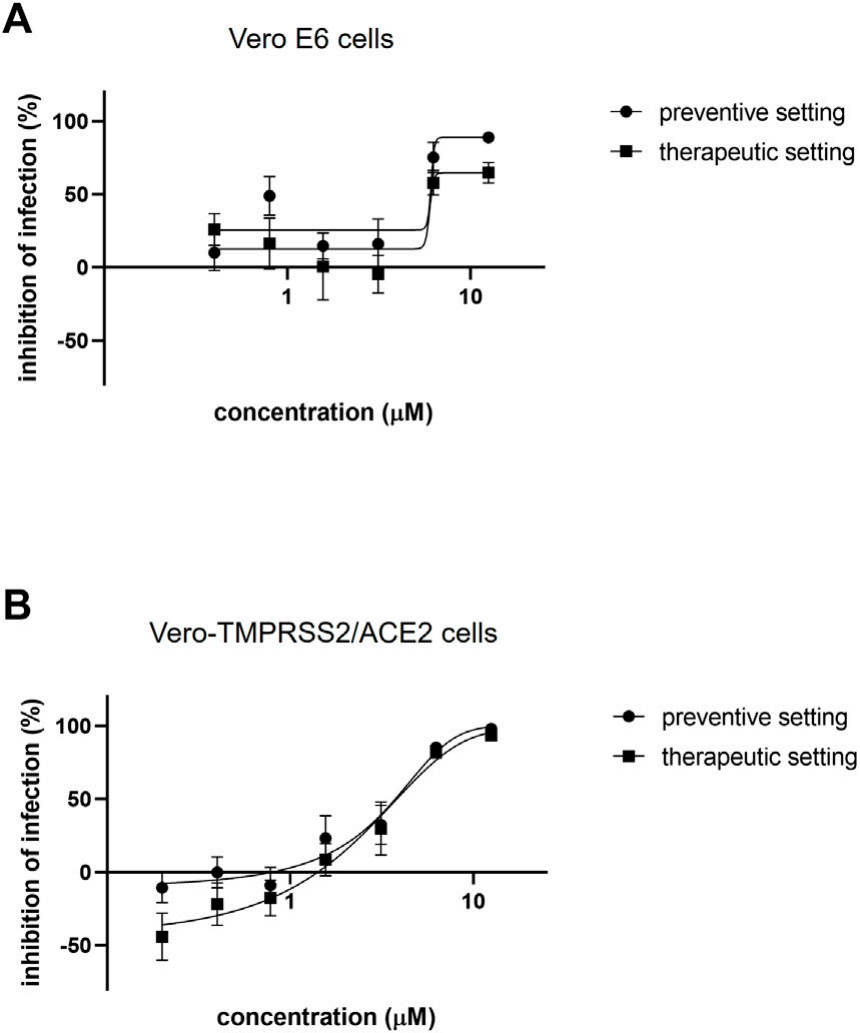
Azelastine is effective against SARS-CoV-2 infection in Vero cell lines. **(A)** Vero E6 or **(B)** Vero-TMPRSS2/ACE2 cells were infected with SARS-CoV-2 B.1.177 simultaneously with or 30 min prior to the addition of 0.4–12.5 µM of azelastine. After 48 h, RNA was extracted from the cell culture supernatant and was quantified by droplet digital PCR **(A)** or qPCR **(B)** analysis. Graphs show inhibition of infection expressed as viral genome count relative to untreated, virus only control (%) and the mean ± SEM from 5 **(A)** or 3 **(B)** biological replicates and 2 **(A)** or 3 **(B)** technical repeats.

**FIGURE 4:**
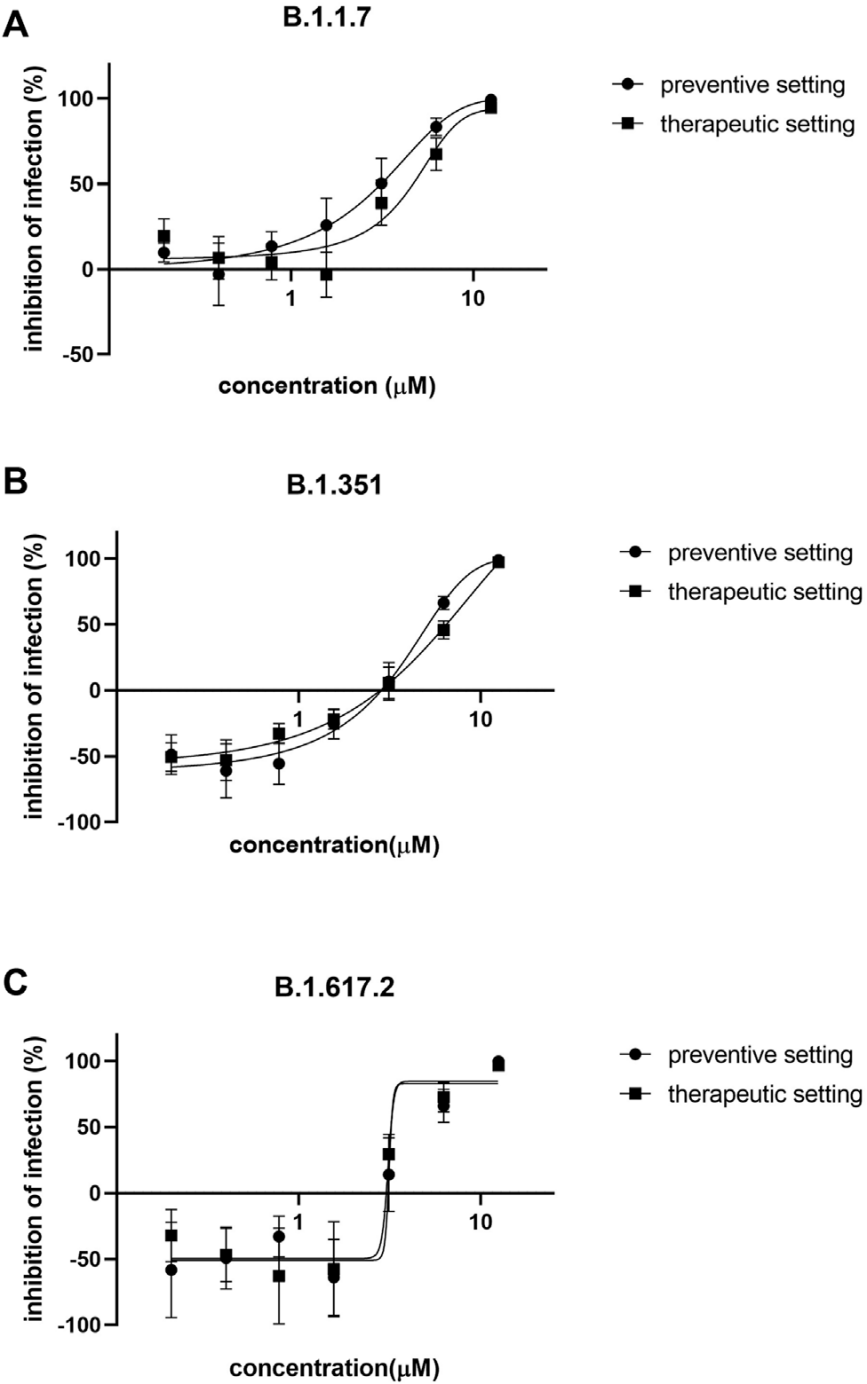
Azelastine is effective against the major variants of concerns in Vero-TMPRSS3/ACE2 cells. Vero-TMPRSS2/ACE2 cells were infected with **(A)** B.1.1.7 **(B)** B.1.351 or with **(C)** B.1.617.2 variant of SARS-CoV-2 simultaneously with or 30 min prior to the addition of 0.4–12.5 µM of azelastine. After 48 h, RNA was extracted from the culture supernatant and was quantified by qPCR analysis. Graphs show percent inhibition of infection based on viral genome counts relative to virus only control expressed as mean ± SEM from 3 independent experiments, each with 3 technical replicates.

Azelastine was first tested for anti-viral effect in a gold standard assay of SARS-CoV-2 infection using the Vero E6 (African green monkey kidney epithelial) cells in the 0.4–12.5 µM concentration range, either with co-administration with the virus or as treatment after viral infection (Figure 3A and Supplementary Figure S2A). Based on a semiquantitative assessment by microscopic examination of cells 48 h post-infection, azelastine was effective in reducing the cytopathic effect in the 3–25 µM concentration range (Supplementary Figure S3). The EC_50_ values of the anti-viral effects based on virus quantification from the culture supernatant were approximately 2.2 and 6.5 µM for the co-administration and treatment settings, respectively (Table 1). The anti-SARS-CoV-2 activity of azelastine was confirmed in a transgenic Vero cell line overexpressing the human serin protease TMPRSS2 and the ACE2 receptor (Figure 3B, Supplementary Figure S2B). In this cell line, azelastine exhibited preventive and therapeutic anti-viral effects against the SARS-CoV-2 virus carrying the spike protein mutation D614G with EC_50_ values of 3.7 and 4 µM respectively. Importantly, in this cell line the anti-viral effect of azelastine was comparable against the major SARS-CoV-2 variants of concern (B.1.1.7, B.1.351 and B.1.617.2) (Table 1; Figure 4 and Supplementary Figure S2C-E) (EC_50_ 2.8–6.5 µM).

**TABLE 1:** 50% effective concentration (EC_50_) of azelastine against various SARS-CoV-2 spike protein mutants and variants in the preventive and therapeutic settings.

SARS-CoV-2 Virus	Cell Line	EC_50_ (µM)	
		Co-Administration	Therapeutic
**D614G**	Vero E6	2.2	6.5
**D614G**	Vero-TMPRSS2/ACE2	3.7	4
**B.1.1.7**	Vero-TMPRSS2/ACE2	2.8	4.3
**B.1.351**	Vero-TMPRSS2/ACE2	5.5	6.5
**B.1.617.2**	Vero-TMPRSS2/ACE2	5.4	4.6

EC_50_ was calculated with log(agonist) vs response variable slope (four parameter).

Azelastine had no effect on the viability of Vero E6 and Vero-TMPRSS2/ACE2 cells below 50 and 25 μM, respectively, as detected by a commercial cytotoxicity assay.

### Anti-SARS-CoV-2 Activity of Azelastine Containing Nasal Spray Using Reconstituted Human Nasal Tissue

Since azelastine is widely used in anti-allergy nasal sprays, we tested one of the commercially available products on reconstituted human 3D nasal tissue (MucilAir™). The tissue samples were first infected with the virus and then treated with the five-fold dilution of the commercial nasal spray solution (0.02% azelastine) for 20 min in every 24 h for 3 days. Microscopic analysis of tissues at 48 and 72 h revealed reduced mucin production in infected cells relative to control cells (no virus or drug treatment) that was prevented in the presence of azelastine (Supplementary Figure S4). No difference in tissue morphology was detected in control and azelastine-treated cells (without virus infection).

Droplet Digital PCR analysis confirmed an effective SARS-CoV-2 infection and viral replication, reaching several thousand copies per microliter by 72 h post-infection in the apical compartment of the tissue inserts (Table 2). Daily 20-min treatment with azelastine (0.02%) drastically reduced the viral particle numbers (>99.9% reduction) at both 48 and 72 h post infection (Table 2).

**TABLE 2:** Viral RNA copy numbers in untreated and azelastine-treated nasal tissues.

	Viral RNA Copy/µl		
	24 hpi	48 hpi	72 hpi
Untreated	0.68	444.67	3,521.33
Allergodil five-fold diluted	0.053 (7.88%)	0.027 (0.01%)	0.05 (<0.01%)

Values represent the average of triplicate samples. Results with azelastine treatment also expressed relative to the untreated infected control (%).

## Discussion

Selected by computational prediction and confirmed by *in vitro* experimental testing, we identified azelastine, an anti-allergy compound broadly available in nasal formulation as a potential anti-COVID-19 remedy.

We pursued a pathway-centric drug repurposing approach to predict drugs with potential anti-COVID-19 activity. Importantly, we did not attempt to reproduce potential anti-viral activities of the selected screening drugs, but instead exploited the finding/observation that similar pathways are affected upon SARS-CoV-2 infection (as shown by the experimental KEGG pathway information) and by drugs with anti-viral activities. In our poly-pharmacological concept, a drug’s mode of action is based on the distribution of protein targets and the biochemical pathways they are part of. We argue this to be a more promising approach compared to conventional single target drug design strategies, particularly in view of the multi-factorial disease phenotype of COVID-19.

Interestingly, we found some overlap with drugs predicted by a complex network approach reported by the Barabási group, which relies on information about human protein binding partners where potential drug candidates are likely to perturb the interactome network relevant for viral infection (Gysi et al., 2021). Although our methodology considers biochemical pathways independent of individual protein interaction events, 15 of the 81 top scoring and prioritized drugs from that study were also identified in our screens.

Several of the drugs predicted in this study, including azelastine have been shown to have activity against coronaviruses *in vitro* (Zhang et al., 2005; Kim et al., 2019; Fan et al., 2020; Gordon et al., 2020; Jeon et al., 2020; Riva et al., 2020; Dittmar et al., 2021; Yang et al., 2021). Furthermore, several of the predicted drugs from our study were or are still being investigated for efficacy in clinical studies (Chiu et al., 2021; Gunst et al., 2021; Kuno et al., 2021; Zhuravel et al., 2021; Quinn et al., 2022).

Among the predicted drugs, we focused on azelastine. This choice was driven by the following: 1., favorable side-effect profile, second generation non-sedating in most individuals, 2., no major systemic effect given the indication to alleviate allergy symptoms topically, 3., broad availability, wide use, low cost; 4., availability in a nasal formulation for potential effect at early phases of viral infection of the nasopharynx (Lythgoe and Middleton, 2020; Dittmar et al., 2021). Additionally, when performing the computational approach with clinically approved SARS-CoV-2 drugs (nirmatrelvir and molnupiravir), there was a high overlap in the top 50 modified pathways between azelastine and nirmatrelvir (29 out of 50), and a lower overlap between azelastine and molnupiravir (11 out of 50).

Here we provide experimental proof that azelastine is effective against SARS-CoV-2 infection in the most widely employed *in vitro* assay system with Vero E6 cells with comparable EC_50_ value (∼6 µM) determined for chloroquine, and remdesivir (7–11 µM) using the same cell line (Jeon et al., 2020). The great disappointment with chloroquine/hydroxychloroquine in clinical efficacy studies raises the concern about the predictive value of such *in vitro* results. More recent data revealed that the choice of cells in SARS-CoV-2 infection assay has great influence on the outcome of drug repurposing testing (Hoffmann et al., 2020; Dittmar et al., 2021). Specifically, hydroxychloroquine was significantly less active against the virus in human respiratory epithelial cells that express the surface protease TMPRSS2 (Ou et al., 2021). Importantly, we confirmed the efficacy of azelastine in a transgenic Vero cell line overexpressing the human serin protease TMPRSS2 and in human respiratory epithelial cells using a highly relevant *in vitro* model, the reconstituted human nasal tissue.

In the transgenic cell line, the anti-SARS-CoV-2 efficacy of azelastine was not only confirmed against the prototype virus that circulated worldwide at the beginning of the pandemic (carrying the spike protein substitution D614G), but also against the subsequently spreading SARS-CoV-2 variants of concern (VOC), including alpha, beta and delta variants. Given that these variants show increased transmissibility and higher virulence as well as the potential to evade vaccine induced immunity, the efficacy against these VOC is of great importance. Furthermore, in the human nasal tissue model we simulated the clinical situation of nasal colonization by SARS-CoV-2 and observed the complete halting of viral propagation even after the first treatment for 20 min with a five-fold diluted (0.02%, 523.5 µM) commercial azelastine-containing nasal spray solution.

The exact molecular mechanism of azelastine’s anti-viral effect is currently not yet delineated and needs further scientific exploration. Interestingly, three independent research groups predicted the interaction of azelastine with the main protease of SARS-CoV-2 called Mpro or 3CLpro (Ghahremanpour et al., 2020; Jain and Mujwar, 2020; Odhar et al., 2020) and one of these three studies also provided experimental evidence for the inhibition of the enzyme in a kinetic activity assay (Ghahremanpour et al., 2020). This potential mode of action warrants further investigation, even if the efficacy of azelastine to inhibit the protease was low -with IC_50_ between 20–100 μM, and recently more effective protease inhibitors have been validated (Owen et al., 2021). Azelastine was also proposed to interfere with the spike glycoprotein/ACE2 interaction through fixing the receptor in a closed formation (Ge et al., 2021).

Azelastine, chemically, belongs to the group of cationic amphiphilic drugs (CAD). This is a pharmacologically diverse group of compounds with various target molecules. Interestingly, CADs are often found by drug repurposing screens against SARS-CoV-2. CADs were shown to cause phospholipidosis (i.e. modulation of lipid processing pathways) in the low µM range, which was demonstrated as the common mechanism in their anti-viral activities (Tummino et al., 2021). Phopholipidosis has been reported as a potential mechanism-of-action responsible for the broad antiviral activity of CADs (as reviewed in (Salata et al., 2017)) including anti-SARS-CoV-2 activity (Vaugeois, 2020; Aimo et al., 2021). However, Tummino et al. concluded in their recent paper that since the anti-viral effect of CADs can only be observed at the high nanomolar/low micromolar concentration (i.e. far above the effective concentration on the original target), it is unlikely that relevant *in vivo* concentrations can be reached without major side effects induced on the original target. Although this may be generally true, azelastine might be considered as an exception for two reasons. First, azelastine is a widely used anti-allergic nasal spray with established safety profile even upon chronic administration and in children above the age of 6 years. Since the clinical indication is topical administration aiming at reducing viral load in the nasopharynx, the relatively high effective dose needed for the antiviral activity could be reached at the target site (nose) without the induction of unwanted systemic effects. Indeed, in case of azelastine we have shown that the 50% effective concentration *in vitro* virus inhibition models is 100–500-fold lower than that of the azelastine concentration in the marketed nasal spray formulation or even in the five-fold diluted nasal spray formulation tested in this study.

Secondly, triggering the original target (i.e. the H1 receptor) is not considered a risk, on the contrary, might also have additional benefit on the outcome of viral infections (Malone et al., 2021). Azelastine and other anti-histamines were shown to exhibit anti-viral effect against various unrelated viruses (Simon, 2003; Vela, 2020). Azelastine is a multifaceted drug; it is best known as a histamine H1 receptor blocker, acting not as an antagonist but as inverse agonist, decreasing H1 receptor constitutive activity (Watts et al., 2019). However, azelastine also has general anti-inflammatory effects, mainly exerted via mast cell stabilization and inhibition of leukotriene and pro-inflammatory cytokine production (Watts et al., 2019). Importantly, mast cells are the main sources of cytokine release that leads to lung damage in SARS-CoV-2, and it has been speculated that mast cell stabilisers may also attenuate pulmonary complications, fatal inflammation and death in COVID-19 (Kritas et al., 2020). Therefore, azelastine’s potential beneficial effects in COVID-19 are expected to be the combination of anti-viral and host-mediated actions.

The major implication of our findings is that a widely available nasal spray formulation containing azelastine might be an immediate solution to prevent and treat nasal colonization with SARS-CoV-2, therefore may have a great impact on the viral spread within the affected person (nasopharynx to lung) as well as within the population. This may be supported by the results of a retrospective cohort analysis of electronic health record data (Bejan et al., 2021), where use of azelastine was associated with lower disease severity. Another association study showed reduced incidence of COVID-19 cases among azelastine-users, and among users of other anti-histamines (Reznikov et al., 2021). Finally, recent data from a clinical study indicate an accelerated virus clearance with the use of an azelastine nasal spray compared to placebo.

## Data Availability

The original contributions presented in the study are included in the article/Supplementary Material, further inquiries can be directed to the corresponding authors.

## Supplementary Material

Supplementary Figure S1 Schematic outline of drug prediction approach. **(A)** Illustration of the first step: Shannon entropy approach for the description of small molecules. The 2D molecular structure is converted into a topological graph (network) in which every atom (node) is attributed with an atom type (feature). The shortest path length is calculated for each and every atom pair (feature pair). The shortest path length distribution N(q) of a given feature pair (i) is converted into a probability p(q) from which the Shannon entropy S_i_ is calculated. The Shannon entropy descriptor SHED of a molecule is therefore the Shannon entropy vector S vector comprising entropy values for the individual feature pairs. **(B)** Illustration of the second step: SHED-based prediction of pathway profiles. The Shannon entropy vector of a molecule is used to screen the DrugBank database for SHED analogs. DrugBank analogs are ranked according to pairwise SHED similarities with the query molecule. Protein targets and the corresponding KEGG pathways of individual DrugBank entries are used to predict the pathway profile of the query molecule (most prevalent/relevant pathways are indicated in red).

Supplementary Figure S2 Azelastine is effective in reducing SARS-CoV-2 viral genome counts *in vitro*. Vero E6 **(A)** or Vero-TMPRSS2/ACE2 cells **(B–E)** were infected with variants of SARS-CoV-2 simultaneously with or 30 min prior to the addition of 0.4 to 12.5 µM of azelastine. After 48 h, RNA was extracted from the cell culture supernatant and was quantified by droplet digital PCR **(A)** or qPCR **(B–E)** analysis. Graphs show mean viral genome counts ±SEM from 5 **(A)** or 3 **(B-E)** biological replicates and 2 **(A)** or 3 **(B-E)** technical repeats.

Supplementary Figure S3 Prevention of SARS-CoV-2 induced cytopathic effect by azelastine. Vero E6 cells were infected with SARS-CoV-2 simultaneously with the addition of 0.4 to 25 µM of azelastine and continued to be cultured without the virus in the presence of the respective concentrations of the drugs. Cytopathic effect was assessed by light field microscopic examination of cultures 48 hours post-infection. **(A)** uninfected (negative) control, **(B)** virus infected (positive) control, **(C)** virus + 3.125 µM azelastine, **(D)** virus + 6.25 µM azelastine, **(E)** virus + 12.5 µM azelastine, **(F)** virus + 25 µM azelastine. **(G)** Scoring system and summary of cytopathic effect in the presence of azelastine (results of two independent experiments).

Supplementary Figure S4 Azelastine blocks viral replication in SARS-CoV-2 infected reconstituted human nasal tissue. MucilAir^TM^ human nasal tissue was infected with SARS-CoV-2 and subsequently treated with five-fold diluted (i.e. final concentration of 0.02% azelastine) nasal spray solution for 20 min in every 24 h for three days. Low resolution microscopic images of cultures after 48 and 72 h of treatment.
