# Supplementary figures and images for: The Anti-Histamine Azelastine, Identified by Computational Drug Repurposing, Inhibits Infection by Major Variants of SARS-CoV-2 in Cell Cultures and Reconstituted Human Nasal Tissue
Source: Front Pharmacol. 2022 Jun 30;13:861295. doi: 10.3389/fphar.2022.861295 (PMC9280057; doi:10.3389/fphar.2022.861295)

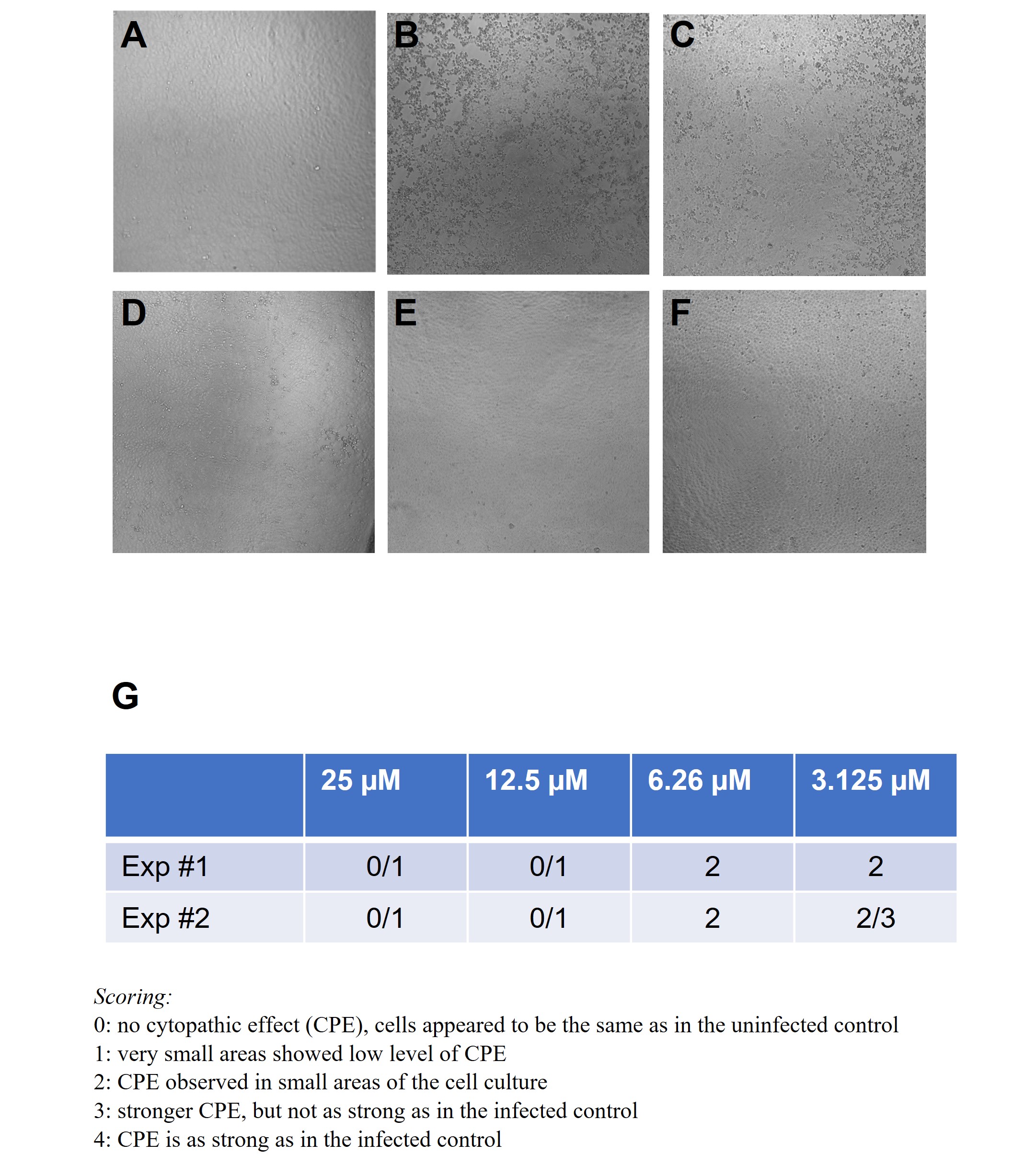

Supplement: Supplementary file 3 [file Image3.jpg]

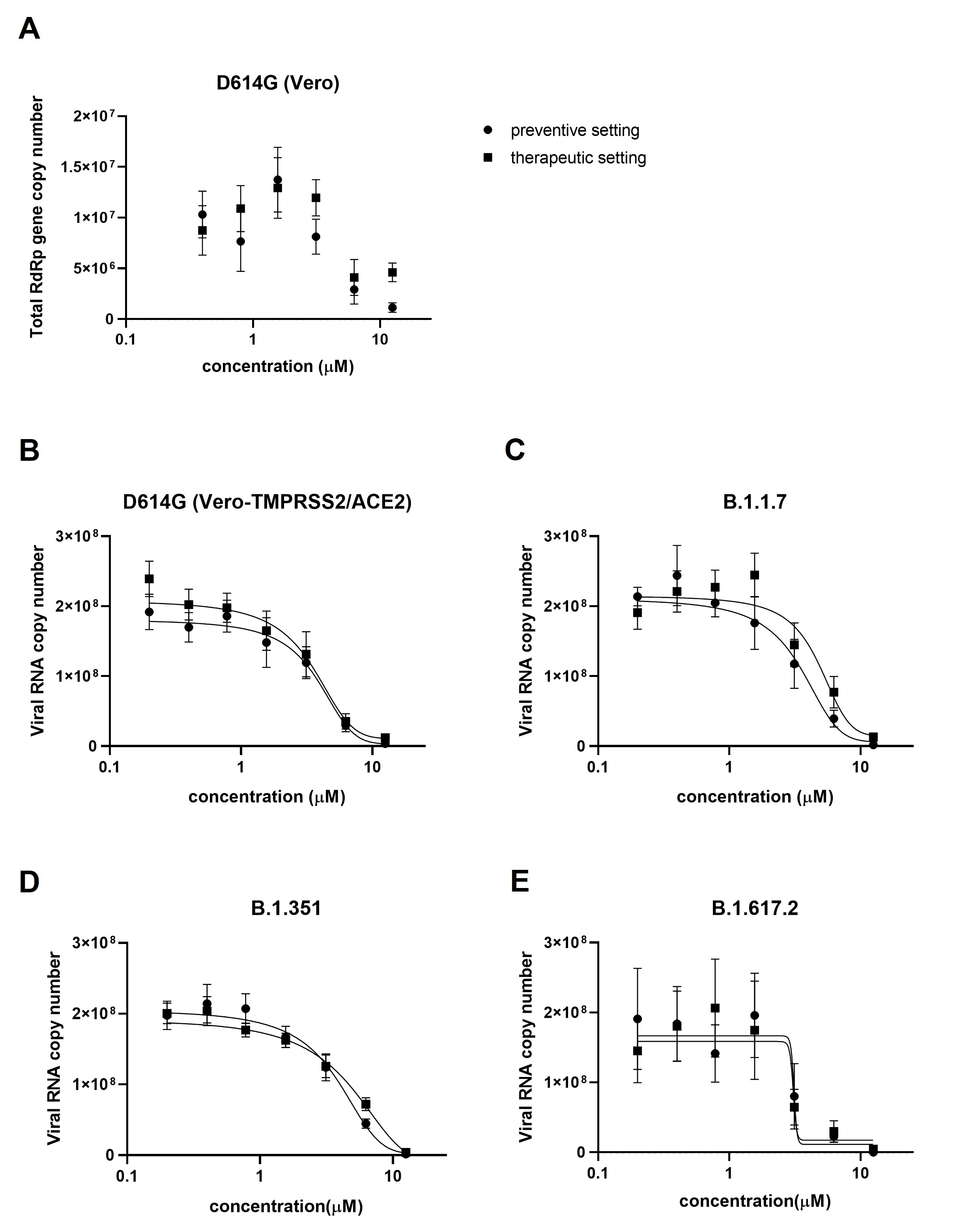

Supplement: Supplementary file 4 [file Image2.jpg]

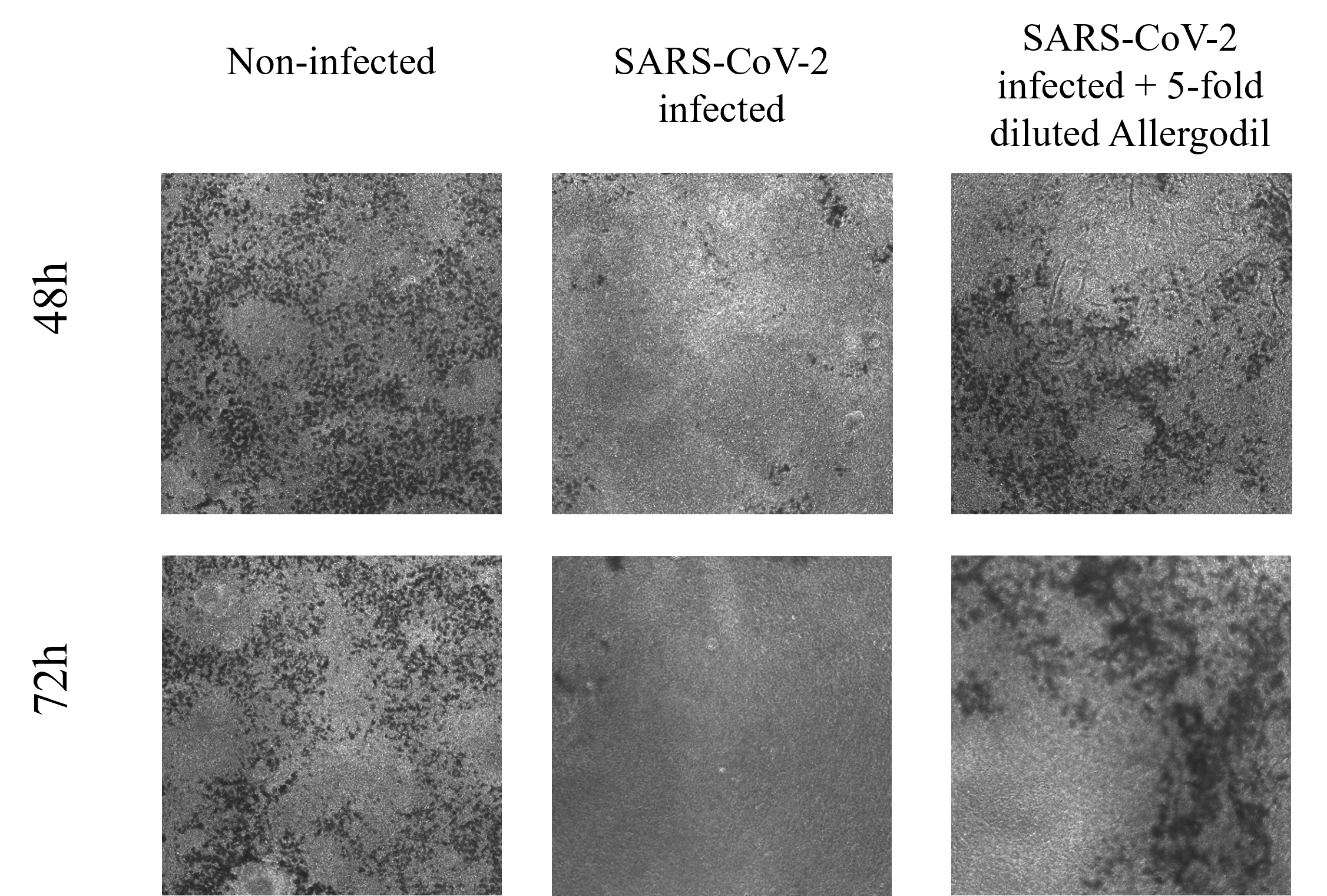

Supplement: Supplementary file 5 [file Image4.tif]

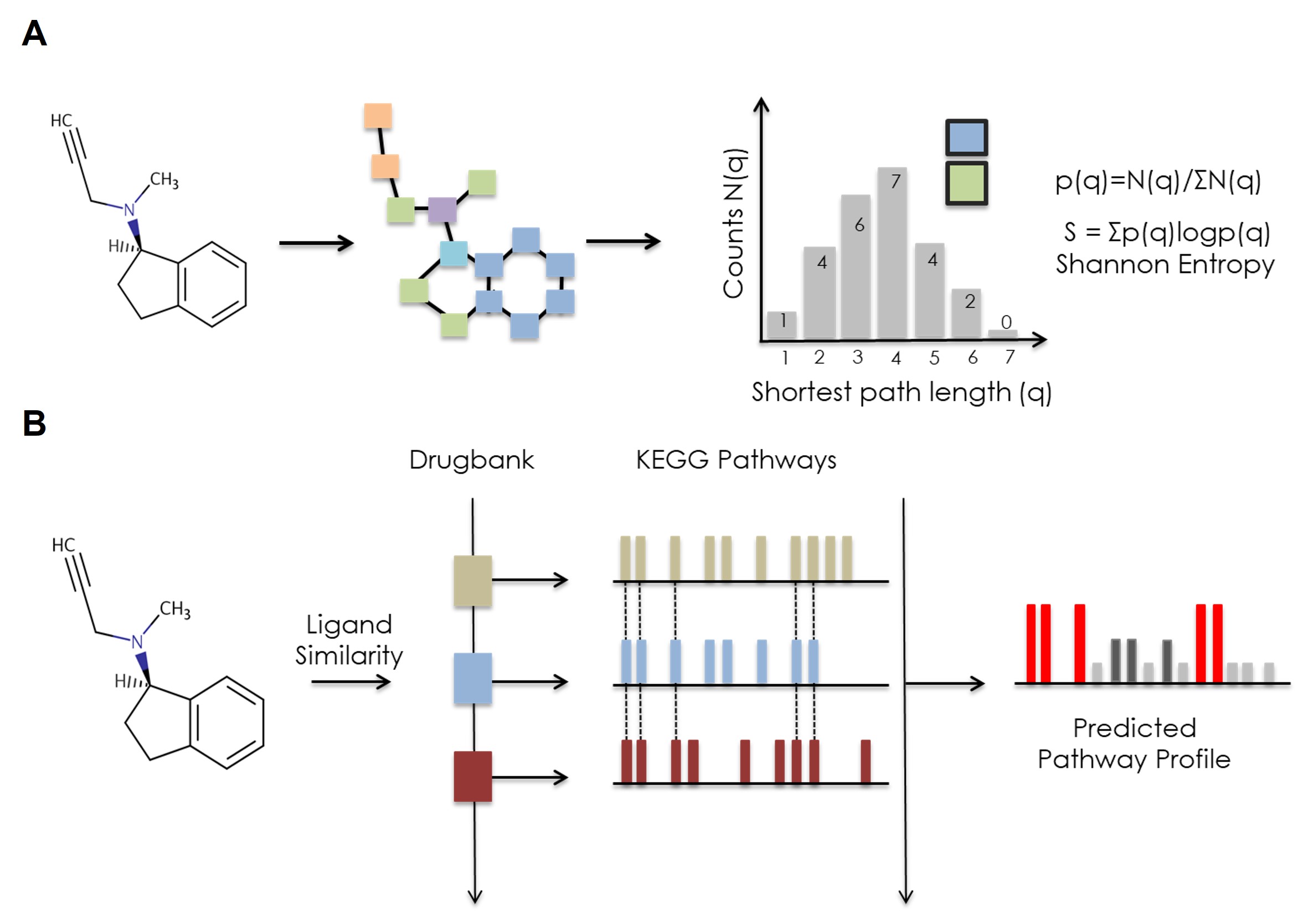

Supplement: Supplementary file 8 [file Image1.jpg]
